# Books and Authors

**Published:** 1909-06

**Authors:** 


### BOOKS AND AUTHORS.

**A Textbook of Medical Chemistry and Toxicology.** By **James W. Holland,** M.D., Professor of Medical Chemistry and Toxicology, Jefferson Medical College, Philadelphia. Second revised edition, octavo of 655 pages, fully illustrated. Philadelphia and London: W. B. Saunders Company, 1908. (Cloth, $3.00 net.)

Four years ago, when the first edition of Professor Holland's work appeared, we gave favorable comment upon it in these columns, not only as to its material but also as to its arrangement, all being especially adapted to the necessities of the student. The section on the urine is the best we have seen both for the student and the practitioner; it is compact, clear, and incisive, telling just enough for both classes of readers. This new edition has been thoroughly revised. We give in the author's own words the more important changes: "While almost every page has been modified for the better, the additions to be especially noted are those relating to the electronic theory; chemical equilibrium; Kjeldahl's method of determining nitrogen; classifications of alkaloids and of protoeins; chemistry of foods and their changes in the body; synthesis of protoeins, and the latest improvements in urinary tests."

Without doubt this book will be recognised as the most practical working textbook for the student and become his companion in the laboratory. Professor Holland is an experienced teacher and a chemist of great knowledge and skill. He has created a book that is entertaining as well as instructive,—an unusual combination in a work on chemistry.

---

**Epoch-Making Contributions to Medicine, Surgery, and the Allied Sciences;** being reprints of those communications which first conveyed Epoch-Making observations to the scientific world, together with biographical sketches of the observers. Collected by **C. M. B. Camac,** M.D., of New York City. Octavo of 435 pages, with portraits. Philadelphia, London. W. B. Saunders Company, 1909. $4.00 net.

A collection of scientific masterpieces like this that have been buried in a mass of literature, therefore inaccessible, is invaluable for reference both to the senior and junior members of the medical profession. The first section deals with antisepsis and contains Lord Lister's famous article on the antiseptic principle of the practice of surgery. The circulation of the blood next appears, and William Harvey's disquisition on the subject is published. Auenbrugger's original treatise on percussion of the chest is next to follow, and then comes auscultation and the stethoscope with Laënnec's writings. Vaccination against smallpox with writings from the immortal Jenner are next given place, then anesthesia, with writings of Morton, Wells, Warren, and Simpson, is set forth; finally, puerperal fever and Oliver Wendell Holmes's essay, brings the volume to a close. Each section, in addition to the writings referred to, contains a portrait of the author, together with a biographical sketch, and a list of his writings.

It must be remembered that the teachings of original observers are of great value even at the present day, that ignorance of these teachings may lead to grave errors; hence the importance of this work in placing in the hands of the profession these classics of medical literature in such available form. The book constitutes an ornamental, as well as useful volume for the table or shelf.

**Practical Dietetics with reference to Diet in Disease,** by **Alida Frances Pattee,** graduate, Boston Normal School of Household Arts. Late Instructor in Dietetics, Bellevue Training School for Nurses, Bellevue Hospital, New York City. Fifth edition. 12mo, cloth. 300 pages. A. F. Pattee, Publisher, Mt. Vernon, N. Y. New York Office, 52 West 39th St., N. Y. (Price, $1.00 net. By mail, $1.10. C. O. D. $1.25.)

One of the remarkable features of Miss Pattee's book is that she is both author and publisher. It has been an unusually successful book, too, being now in its fifth edition. It is in use in the army hospitals, and is adopted as a textbook by the state boards of examiners for nurses in many states. We have regarded it from the first as one of the best authorities on dietetics extant. It contains the diet lists for various diseases and tells what foods to avoid in each. It also instructs as to the proper food for infants, based on the recommendations of experienced physicians. It is a reference book on diet for the physician and teaches the student what cannot be learned in the classroom. In short, it is a book for physicians, student, nurse, hospital and home.

---

**Bacterial Food Poisoning.** A Concise Exposition of the Etiology, Bacteriology, Pathology, Symptomatology, Prophylaxis, and Treatment of So-Called Ptomaine Poisoning. By **Professor Dr. A. Dieudonné,** Munich. Authorized translation, edited, with additions, by **Dr. Charles Frederick Bolduan,** Bacteriologist, Research Laboratory, Department of Health, City of New York. 8vo, 125 pages. New York: E. B. Treat & Co., Medical Publishers, 241-243 West 23d Street. (Cloth, prepaid, $1.00 net.)

It is well to have food poisons pointed out to the profession in such an accessible form as this book presents. This monograph deserves the careful study of physicians, and it contains much that would prove interesting and instructive to the laity. Poisoning due to eating the meat of diseased animals is often so subtle as to escape observation as to cause until sickness therefrom is well under way. Bacteriology here often points out the way when otherwise the attendant would wade in doubt, possibly until too late to effect cure. When meat is eaten that has undergone putrefactive changes, the poison is more active—ptomaines—and the case may prove fatal in a few hours. Next to meat in importance comes fish and molluscs, and then canned foods play an important part in causing poisoning. The monograph should have a wide circulation as it deals scientifically with questions of great importance.

---

**Blood Examination in Surgical Diagnosis.** A Practical Study of Its Scope and Technic. By **Ira S. Wile,** M.D., New York. Duodecimo; 161 pages; 35 illustrations and 1 double-page colored plate. New York: Surgery Publishing Company, 1908. Cloth, price $2.00; oil cloth for laboratory use, $2.50; de luxe ooze leather, price $3.00.

The special object of this book, as its title indicates, is to point out the value of blood examination as an aid to surgical diagnosis. Literature has been examined with reference to its surgical value in a hematological sense, facts have been grouped as far as they apply and the result is this work, which might properly bear the name of surgical hematology. But this is not all: the author has done some original work that is herein incorporated. The surgical interpretation of the total leukocyte count is new, and so is the differential count. The publisher sends out a "review" from which we extract the following description, stripped of its somewhat fulsome praise.

Lacking in hematological dogmatism it abounds in a surgical conservatism that makes it a safe authority to follow. The consideration of anti-operative, operative and post-operative conditions affecting the blood is logical and well arranged. The book is an example of the printer's art and the bookbinder's ability. The topography is clear and attractive, and the marginal notes in red are useful. The double-page colored plate shows six blood pictures and in addition twenty-nine illustrations of the various types of cells as they appear with the Jenner stain. While these will serve to interpret, the numerous other blood pictures printed only in black, have added to the value of the book.

---

**Primary Studies for Nurses:** A Textbook for First Year Pupil Nurses. By **Charlotte A. Aikens,** formerly Superintendent of Columbia Hospital, Pittsburg, and of the Iowa Methodist Hospital, Des Moines. 12 mo. of 435 pages, illustrated. Philadelphia and London: W. B. Saunders Company, 1909. Cloth, $1.75 net.

This book is intended especially for first-year pupil nurses, yet is appropriate for even more advanced students. It contains just enough on the topics of anatomy and physiology to be suggestive of those subjects for more advanced grades. The definitions are simple that they may come within the comprehension of the novitiate. Hygiene is the subject of the second section, general hygiene being presented first, then personal hygiene. The third section deals with bacteriology, the fourth with therapeutics and materia medica, the fifth with dietetics, the sixth with invalid cookery, while the seventh section presents questions for self-examination and review. Thus it will be observed that primary topics are handled for the benefits of beginners. Miss Aikens is a master of her subject and her book is the best of its class.

---

**A Textbook of Materia Medica, Pharmacology and Therapeutics.** By **George F. Butler,** M.D., Professor and Head of the Department of Therapeutics and Professor of Preventive and Clinical Medicine, Chicago College of Medicine and Surgery, Medical Department Valpariso University. Sixth edition, revised and enlarged. Octavo of 708 pages. Philadelphia and London: W. B. Saunders Company, 1908. (Cloth, $4.00 net, half-morocco, $5.50 net.)

That this textbook enjoys a full measure of popularity is attested by the fact that six editions have been put forth since

1896. It is a book for both physician and student, its arrangement being such as to suit both classes of readers or searchers. All the official and some of the unofficial remedies are mentioned. This edition follows the same lines as those that have preceded it. The book is free from useless verbiage, or obsolete material, practical rather than theoretical, and is well to the fore in all that relates to materia medica, pharmacology, and therapeutics. Butler is of the opinion that the present tendency toward agnosticism in therapeutics and to nostrum prescribing are due to the neglect of therapeutics in the medical schools—a condition from which he seeks to reclaim the profession.

---

I. **Atlas and Epitome of External Diseases of the Eye.** By Professor **Dr. O. Haab,** of Zurich. Edited, with additions, by **George E. deSchweinitz,** M.D., Professor of Ophthalmology, University of Pennsylvania. Third revised edition. With 101 colored lithographic illustrations on 46 plates and 244 pages of text. Philadelphia and London: W. B. Saunders Company, 1909. Cloth, $3.00 net.

II. **Atlas and Epitome of Ophthalmoscopy and Ophthalmoscopic Diagnosis.** By **Professor Dr. O. Haab,** of Zurich. Edited, with additions, by **George E. deSchweinitz,** M.D., Professor of Ophthalmology, University of Pennsylvania. Second revised edition. With 152 colored lithographic illustrations and 94 pages of text. Philadelphia and London: W. B. Saunders Company, 1909. $3.00 net.

I. These two volumes, being each the counterpart of the other, may be reviewed together with propriety. The first, on ophthalmoscopy or the use of the ophthalmoscope in diagnosis, is a most valuable exposition of the subject, and should be mastered by every beginner. The text is clear, incisive, and brief, making it easy to acquire the necessary knowledge of the technic of the instrument to examine the eye-ground, in a short time. The colored plates of the atlas are exact portrayals of pathologic conditions, after giving the normal eye-ground, and are 87 in number, giving 152 illustrations. They deserve a word or two of commendation for, although we mentioned their superior quality when noticing the first edition, we desire to emphasise their excellence again. It is doubtful if ever before have such splendid portrayals of the interior structures of the eye been published in book form. The publishers are entitled to great credit for the superb manner in which their work is done.

II. Unlike the previous volume, in this one the illustrations are distributed throughout the text, which enables the reader to examine the disease which he is considering. There are 101 illustrations in this book depicting the most important diseases and injuries of the eye. It is remarkable how well the artist has pictured these affections, the approach to perfection, indeed, being very close; to examine these reproductions in connection with the text is the next thing to seeing the patients themselves in the clinic. Every one interested in ophthalmology, be he student or practitioner, will be sure to appreciate the efforts of author, artist, and publishers to bring out this work on such a high plane of perfection. In the first American edition of these books was given notice in detail, hence further remarks at this time would seem inappropriate if not unnecessary.

---

**Anatomie Und Mechanismus Der Skoliose.** By **Dr. Carl Nicoladoni,** Graz. Illustrated. Berlin: Urban & Schwarkenberg.

This monograph consists of sixty pages of text, and an atlas containing thirty-seven plates on which are fifty-four engravings. The illustrations exhibit all phases of the bony conditions pertaining to scoliosis. They are superbly drawn and illumine the text in a satisfactory manner, bringing out the anatomy, of the disease and showing its mechanism. It is desirable that an English translation of the text should be made in order that the monograph may become available to all interested in the disease who may not read German.

---

**Transactions of the Sixth Annual Conference of State and Territorial Health Officers with the United States Public Health and Marine Hospital Service,** held at Washington, D. C., April 27, 1908.

This book contains an interesting report of conferences by health officers throughout the country with the surgeon-general of the Public Health and Marine Hospital Service, for the purpose of bringing about concert of action regarding interstate quarantine regulations and other public health measures. It contains a verbatim report of the work of the conference, and should be read by every state and territorial health officer. It also, would be well if local health officers were supplied with copies of the book along our great rivers and other waterways. Too much intelligence can not be gained regarding the spread of contagious diseases.

---

**New and Nonofficial Remedies.** Articles which have been accepted by the Council on Pharmacy and Chemistry of the American Medical Association, prior to January, 1909. Chicago: Press of the American Medical Association, 103 Dearborn Avenue. (Paper, 25c; cloth, 50c.)

This small volume contains a description of the medicinal substances that have been approved by the council on pharmacy and chemistry of the American Medical Association. The contents embrace only those remedies examined prior to January 1, 1909. There is no occasion to "review" the book in the ordinary acceptation of the term. The council sends with the work a statement of its purpose from which we extract a few sentences.

This is the first regular edition of the Annual New and Non-official Remedies. Instead of adhering strictly to an alphabetic arrangement a classification has been adopted which permits an easy comparison of remedies of similar origin and properties. Mixtures are to be found in the appendix and a number of non-proprietary preparations have been added which, for various reasons, have not been admitted to the pharmacopeia. The descriptions in the appendix have been made as brief as possible and the articles are classified under the names of the manufacturers.

The descriptions of processes of preparations, chemical and physical, and of the physiologic action contain much information which can not fail to be of immense value both to physicians and to pharmacists. Over 200 different remedies are described, and after mastering the pharmacopeia the practitioner and the student should become thoroughly familiar with this presentation of the newer materia medica.

---

**Refraction and How to Refract,** including sections on Optics, Retinoscopy, fitting of Spectacles and Eye Glasses. By **James Thorington,** A.M., M.D., professor of diseases of the eye in the Philadelphia Polyclinic and College for graduates in medicine, etc. Fourth edition. Two hundred and twenty illustrations, thirteen being in colors. Philadelphia: P. Blakiston's Son & Company. 1909. (Price, cloth $1.50.)

This author is an experienced refractionist, and is an expert with reference to all that pertains to the subject. Four editions in ten years on such a topic speak emphatically as to its value. It is so important that eyes shall be fitted properly when glasses are required, that we need scarcely discuss the matter, particularly as the laity also understands its importance pretty well. The illustrations, many being original, deserve mention as they are very helpful in explaining the text. Thorington has carefully revised the reading matter in this edition, and incorporated an appendix that further develops information relating to refraction. As now constituted, it is doubtful if any work equals it on the subject with which it deals.

---

**Theory and Practice of Infant Feeding** with notes on development, by **Henry Dwight Chapin,** A.M., M.D. Professor of Diseases of Children. New York Post-Graduate Medical School and Hospital. Third edition, revised, with illustrations. New York: William Wood & Co. 1909.

Chapin is an experienced, scientific pediatrist, and has an especial gift in the direction of infant feeding. He has created a book that is second to none on this subject. The two editions preceding this were issued before the science and art of infant feeding had reached its present stage. Chapin, himself, states in effect that methods formerly deemed unscientific are now acknowledged to be in accord with natural biological laws; whereas, some methods formerly employed have been proven to be inadequate, if not improper. Hence, in this edition the chapters on practical feeding have been rewritten, while sections on the principles of top milks and the standardisation of gruels have been added. The work now stands at the head of the list on the feeding of infants.

---

**Seven Hundred Surgical Suggestions.** Practical Brevities in Surgical Diagnosis and Treatment. By **Walter M. Brickner,** B.S., M.D., Assistant Adjunct Surgeon, Mount Sinai Hospital, New York; Editor-in-Chief, American Journal of Surgery, Eli Moschcowitz, A.B., M.D., Assistant Physician, Mount Sinai Hospital Dispensary, New York, and Harold M. Hays, M.A., M.D. Third Series. Duodecimo; 153 pages. New York: Surgery Publishing Co., 92 William Street. Price, semi de luxe, $1.00; full library de luxe ooze leather, gold edges, $2.25.

Though this book is small it contains much that is useful. It is of value, in particular, for the junior practitioner and its size is such as to permit it to be carried in the pocket. The topics are classified under marginal headings in red, making any desired subject easy of access. It would be difficult to improve upon the mechanical make-up of the book when its purpose is taken into consideration. If every book, large or small, presented as much material of real value as this one, medical libraries would contain more scientific truth than they do at present.

